# Community pharmacists and outpatient antimicrobial stewardship: evidence, uncertainties, and future directions

**DOI:** 10.3389/frhs.2026.1797660

**Published:** 2026-07-16

**Authors:** Jin Jung, Seonju Jung, Chiranjeev Sanyal

**Affiliations:** College of Pharmacy, Dalhousie University, Halifax, Nova Scotia, Canada

**Keywords:** antibiotic duration, antimicrobial resistance, antimicrobial stewardship, community pharmacy, outpatient prescribing

## Introduction

Antimicrobial resistance (AMR) has emerged as one of the most significant global public health threats of the twenty-first century. The World Health Organization has identified AMR as a major challenge that threatens the effectiveness of modern medicine and increases morbidity, mortality, and healthcare costs (1). Excessive and inappropriate antibiotic use remains one of the principal drivers of resistance development (2).

Outpatient settings account for the majority of antibiotic prescribing and consumption in many high-income countries. Estimates suggest that approximately 70%–90% of antibiotic use occurs in primary care and community settings, where respiratory tract infections, urinary tract infections, and skin infections are commonly treated (3, 4). In the United States, nearly one-third of outpatient antibiotic prescriptions may be unnecessary (4), while considerable variation in prescribing practices has been documented internationally (5, 6).

Community pharmacists occupy a potentially important position within outpatient antimicrobial stewardship because they are highly accessible healthcare professionals with expertise in medication management. Several studies have suggested that pharmacists may contribute to antibiotic optimization through patient education, medication review, point-of-care testing, and, in some jurisdictions, prescribing activities (7).

Nevertheless, the literature presents a more complex picture than is sometimes reflected in policy discussions. Evidence supporting pharmacist-led stewardship frequently derives from specific healthcare systems, selected patient populations, and relatively uncomplicated infections. Considerable variation exists in pharmacist prescribing authority, reimbursement structures, access to medical records, and integration within primary care teams (7, 8).

Furthermore, the growing evidence supporting shorter antibiotic durations for selected infections has introduced new questions regarding the role of pharmacists in promoting evidence-based treatment decisions. While shorter courses appear effective for several uncomplicated infections (9, 10), uncertainty remains regarding patient selection, implementation, and applicability to more complex clinical scenarios.

The present paper differs from previous discussions of pharmacist-led stewardship in three important respects. First, it integrates evidence regarding antibiotic treatment duration with broader antimicrobial stewardship activities. Second, it critically examines areas of uncertainty and conflicting evidence alongside evidence of benefit. Third, it considers how healthcare system differences may influence the applicability of stewardship models across jurisdictions.

Rather than advocating a uniform expansion of pharmacist prescribing authority, this paper examines the evidence supporting pharmacist involvement in outpatient antimicrobial stewardship and discusses the circumstances under which such interventions may contribute to improved antibiotic use.

## Community pharmacists and outpatient stewardship

Community pharmacists possess several characteristics that may support antimicrobial stewardship activities. Pharmacists are often the most accessible healthcare professionals, frequently encounter patients seeking advice regarding infectious symptoms, and maintain longitudinal medication records that can identify recurrent antibiotic use or treatment failures (11).

Several potential stewardship functions have been identified:

- optimization of antibiotic dispensing;
- identification of inappropriate prescribing;
- patient education regarding antibiotic use;
- adherence counseling;
- monitoring adverse effects;
- participation in point-of-care testing programs;
- collaborative prescribing within defined protocols.

However, the contribution of these activities may depend heavily upon local healthcare infrastructure, professional regulation, and access to clinical information. Consequently, evidence obtained in one healthcare system may not necessarily be generalizable to another.

## Antibiotic duration as a stewardship issue

Traditional advice encouraging patients to complete antibiotic courses has increasingly been questioned. Several studies have demonstrated that prolonged antibiotic exposure may increase selective pressure for resistance without improving clinical outcomes (12).

Palin et al. (13) reported that longer antibiotic courses for common infections were associated with increased infection-related complications and greater antibiotic exposure without clear clinical advantages. Furukawa et al. (9) demonstrated that five-day treatment courses for community-acquired pneumonia frequently produced outcomes comparable to longer regimens.

Similar findings have been reported for uncomplicated urinary tract infections, sinusitis, and selected skin infections (14). Contemporary stewardship frameworks increasingly emphasize minimizing unnecessary antibiotic exposure (15).

Nevertheless, shorter treatment durations are not universally appropriate. Patients with immunosuppression, severe infection, chronic comorbidity, treatment failure, or resistant pathogens may require individualized treatment approaches. Clinical judgment therefore remains essential.

Community pharmacists may contribute to these discussions through patient education, adherence counseling, symptom assessment, and referral when appropriate. However, pharmacists generally lack access to physical examination findings, imaging, and laboratory data, which may limit their ability to independently determine treatment duration in more complex cases.

## Evidence supporting pharmacist-led interventions

Several studies have reported favorable outcomes associated with pharmacist involvement in outpatient antimicrobial stewardship, although the strength and generalizability of this evidence vary considerably.

A systematic review by Wu et al. (7) identified multiple antimicrobial stewardship interventions delivered by community pharmacists, including prescribing for minor infections, point-of-care testing, patient education, and medication review. The review concluded that pharmacist-led interventions frequently improved guideline adherence and patient satisfaction, although the authors emphasized that the overall quality of evidence remained moderate and that most studies involved uncomplicated infections.

Management of uncomplicated urinary tract infections has received particular attention. The R(x)OUTMAP study demonstrated clinical cure rates exceeding 88%–90% among patients managed by pharmacists for uncomplicated urinary tract infections, with low rates of treatment failure and adverse events (16). Similar findings have been reported in provincial prescribing programs in Canada and minor ailment services in the United Kingdom (17, 18).

Point-of-care testing represents another area of interest. Papastergiou et al. (19) found that pharmacist-directed group A streptococcal testing improved diagnostic accuracy and reduced unnecessary antibiotic use among patients presenting with sore throat symptoms. Thornley et al. (20) similarly reported favorable outcomes following community pharmacy streptococcal testing services in the United Kingdom.

Patient education may also contribute to stewardship efforts. Community pharmacists routinely discuss adherence, adverse effects, expected symptom duration, and appropriate antibiotic use. Langford and Morris (12) argued that pharmacists may play an important role in correcting misconceptions surrounding the traditional advice to “finish the entire course” regardless of clinical improvement.

Several studies have suggested that pharmacist interventions may reduce healthcare utilization. Stewart et al. (21) reported that pharmacy-based management programs for uncomplicated infections reduced demands on primary care services. Similar observations have been reported in pharmacist prescribing programs in Canada and Scotland (16, 22).

However, interpretation of these findings requires caution. Most studies involve carefully selected patients with uncomplicated infections, highly trained pharmacists, structured protocols, and supportive healthcare systems. Few investigations have evaluated long-term outcomes such as antimicrobial resistance patterns, hospital admissions, or population-level prescribing changes.

Furthermore, positive outcomes frequently originate from pilot programs or early implementation studies that may not fully reflect routine clinical practice. Consequently, existing evidence may support the potential effectiveness of pharmacist-led stewardship interventions while simultaneously highlighting the need for additional large-scale evaluations.

## Areas of uncertainty and conflicting evidence

Despite increasing interest in pharmacist-led antimicrobial stewardship, several important uncertainties remain. First, evidence regarding the direct effect of pharmacist interventions on antimicrobial resistance is limited. Most studies evaluate short-term clinical outcomes, patient satisfaction, or prescribing rates rather than resistance development (7). Because antimicrobial resistance evolves over extended periods and is influenced by multiple factors, establishing causal relationships between pharmacist interventions and resistance patterns remains challenging. Second, the available evidence is heavily weighted toward uncomplicated infections such as urinary tract infections, pharyngitis, and minor skin infections. Limited data exist regarding more complex presentations, older adults with multiple comorbidities, immunocompromised individuals, or patients with recurrent infections (16). Third, concerns have been raised regarding protocol-driven prescribing. Expanded prescribing authority may improve access to care, but it could also increase antibiotic exposure if diagnostic uncertainty leads to defensive prescribing practices. Some investigators have cautioned that greater access to antibiotics, without sufficient stewardship oversight, could unintentionally contribute to inappropriate prescribing (23).

Diagnostic uncertainty remains a significant concern in community practice. Pharmacists generally lack access to physical examinations, imaging studies, microbiological testing, and comprehensive medical records. Atypical presentations, early serious infections, and evolving clinical conditions may therefore be difficult to identify. Several studies examining prescribing variation among physicians demonstrate substantial differences in antibiotic use despite similar clinical presentations (5, 6). Similar variation may also occur among pharmacists if standardized competencies and stewardship training are not consistently implemented. Patient expectations further complicate stewardship efforts. Public demand for antibiotics continues to influence prescribing decisions in primary care settings (24). Pharmacists practicing in busy retail environments may experience pressure to satisfy patient expectations, particularly where clinical assessment time is limited.

The evidence regarding shorter antibiotic courses also remains nuanced. Although shorter regimens appear effective for many uncomplicated infections, important exceptions exist. Patients with severe infections, immunosuppression, abscesses, resistant pathogens, or delayed clinical response may require individualized treatment durations (14). Consequently, antibiotic duration decisions often require clinical judgment that extends beyond standardized protocols. These uncertainties do not negate the potential value of pharmacist-led stewardship. Instead, they suggest that implementation should occur cautiously, with appropriate governance structures and ongoing evaluation.

## Healthcare system differences and generalizability

The international literature on pharmacist-led antimicrobial stewardship is derived primarily from Canada, the United Kingdom, Australia, and the United States. While these countries share similarities as high-income healthcare systems, important structural differences limit the transferability of findings. Canada has experienced substantial expansion of pharmacist prescribing authority, although considerable provincial variation exists. Alberta pharmacists possess broader prescribing authority than pharmacists in many other provinces, potentially influencing study outcomes (16). The United Kingdom has implemented nationally coordinated pharmacy initiatives, including Pharmacy First programs and independent prescribing pathways (18). These services operate within the National Health Service and often benefit from centralized funding and integrated care pathways. Australia has adopted selected pharmacist prescribing initiatives and pilot programs, although implementation remains variable across states and territories (8). The United States presents a more fragmented environment characterized by state-level regulation, variable collaborative practice agreements, and diverse reimbursement models (25).

Differences in electronic health records, reimbursement systems, workforce capacity, prescribing authority, and access to diagnostic testing may substantially influence the effectiveness of stewardship interventions. Consequently, findings from one jurisdiction cannot necessarily be generalized to another. The successful implementation of pharmacist-led antimicrobial stewardship may depend upon local regulatory frameworks, healthcare infrastructure, workforce training, and relationships with other healthcare providers. The present discussion therefore applies primarily to high-income countries with established community pharmacy infrastructure. Extrapolation to low- and middle-income settings should be undertaken cautiously because healthcare delivery models, antibiotic access, and regulatory systems may differ substantially (2).

## Implementation challenges

Even where pharmacists possess prescribing authority, multiple barriers may limit the implementation of stewardship activities. Workload remains one of the most frequently cited challenges. Community pharmacists often manage high prescription volumes, vaccination programs, medication reviews, and administrative responsibilities simultaneously. Limited time may reduce opportunities for comprehensive assessment and patient counseling. Workforce shortages further constrain service delivery. Community pharmacies in many jurisdictions face recruitment difficulties, increasing prescription volumes, and expanding clinical responsibilities. Without additional staffing resources, implementation of stewardship activities may prove difficult.

Documentation and communication challenges are also common. Pharmacists frequently lack access to electronic medical records, laboratory results, and previous clinical assessments. Inadequate information sharing may contribute to fragmented care and duplicated treatment. Financial barriers remain substantial. Many healthcare systems reimburse product dispensing but provide limited compensation for assessment, follow-up, or stewardship activities. The absence of sustainable funding models may reduce the feasibility of expanded services. Professional relationships may also influence implementation. Collaborative relationships between pharmacists and physicians facilitate stewardship activities, whereas professional tensions or unclear responsibilities may hinder integration.

Patient expectations represent another important consideration. Public misunderstanding regarding antibiotics remains widespread, and pharmacists may encounter pressure to provide antibiotics even when clinical indications are uncertain (24). These implementation barriers suggest that pharmacist-led stewardship cannot be considered solely a professional scope issue. Successful programs may require coordinated changes involving reimbursement, training, information systems, and interprofessional collaboration.

## Policy considerations

Several policy approaches have been proposed to support pharmacist participation in antimicrobial stewardship. However, the available evidence does not support a single universal model. Expanded prescribing authority has received considerable attention. Proponents argue that pharmacist prescribing may improve access to care and reduce delays in treatment. Nevertheless, the effectiveness and safety of expanded authority likely depend upon standardized protocols, appropriate training, and effective referral pathways.

Standardized stewardship training represents another potential strategy. Infectious disease management, diagnostic reasoning, and antimicrobial stewardship competencies vary considerably among jurisdictions. Certification programs and continuing education may improve consistency, although evidence regarding their effectiveness remains limited. Reimbursement reforms have also been proposed. Compensation for assessment, point-of-care testing, follow-up, and documentation could increase the feasibility of stewardship services. However, the economic implications of these models require further study.

Integrated care models may offer additional opportunities. Shared electronic health records, collaborative practice agreements, and communication pathways could improve continuity of care and support stewardship activities. Point-of-care testing may enhance diagnostic accuracy in selected clinical situations, although implementation costs, training requirements, and test performance characteristics should be considered. Rather than representing definitive solutions, these policy options may be viewed as potential approaches whose effectiveness will likely depend on local healthcare contexts.

## Future research priorities

Several research gaps continue to limit understanding of pharmacist-led antimicrobial stewardship. First, studies evaluating long-term antimicrobial resistance outcomes are needed. Most existing investigations examine short-term clinical endpoints rather than resistance patterns. Second, comparative studies involving different healthcare systems would help identify which organizational factors contribute to successful implementation. Third, economic evaluations are needed to determine the cost-effectiveness of pharmacist-led stewardship services, including prescribing, point-of-care testing, and follow-up activities. Fourth, research involving complex patients, older adults, and individuals with multiple comorbidities would improve understanding of intervention safety and effectiveness beyond uncomplicated infections. Fifth, implementation science approaches could identify barriers and facilitators associated with integrating stewardship activities into routine practice. Finally, patient perspectives remain relatively understudied. Understanding patient expectations, satisfaction, and acceptance of pharmacist-led services may inform future program design.

## Conclusion

Community pharmacists may contribute meaningfully to outpatient antimicrobial stewardship through patient education, medication management, point-of-care testing, and selected prescribing activities. The available evidence suggests potential benefits for uncomplicated infections and selected clinical scenarios. However, important uncertainties remain. Much of the literature derives from specific healthcare systems, uncomplicated patient populations, and pilot programs. Evidence regarding long-term antimicrobial resistance outcomes, broader implementation, and healthcare system transferability remains limited. Recent evidence supporting shorter antibiotic durations for selected infections offers an opportunity to reconsider the pharmacist's role in promoting evidence-based antimicrobial use. Nevertheless, treatment decisions frequently require individualized clinical judgment, particularly among patients with severe illness, comorbidities, or diagnostic uncertainty.

The contribution of community pharmacists to antimicrobial stewardship may therefore depend less upon professional scope alone and more upon the availability of appropriate training, governance structures, collaborative relationships, and healthcare system support. Future investigations that evaluate long-term outcomes, implementation strategies, and healthcare system differences may help clarify the circumstances under which pharmacist-led stewardship can most effectively contribute to antimicrobial resistance mitigation and improved patient care.

## References

[1] World Health Organization. Antimicrobial Resistance: Fact Sheet. Geneva: WHO (2023).

[2] Sulis G Adam P Nafade V Gore G Daniels B Daftary A. Antibiotic prescription practices in primary care in low- and middle-income countries: a systematic review and meta-analysis. PLoS Med. (2020) 17:e1003139. 10.1371/journal.pmed.1003139 32544153 PMC7297306

[3] European Centre for Disease Prevention and Control. Antimicrobial Consumption in the EU/EEA (ESAC-Net): Annual Epidemiological Report for 2023. Stockholm: ECDC (2024).

[4] Hersh AL King LM Shapiro DJ Hicks LA Fleming-Dutra KE. Unnecessary antibiotic prescribing in US ambulatory care settings, 2010–2015. Clin Infect Dis. (2021) 72(1):133–7. 10.1093/cid/ciaa667 32484505 PMC9377284

[5] Butler CC Hood K Verheij T Little P Melbye H Nuttall J. Variation in antibiotic prescribing and its impact on recovery in patients with acute cough in primary care: prospective study in 13 countries. Br Med J. (2009) 338:b2242. 10.1136/bmj.b2242 19549995 PMC3272656

[6] Kasse GE Humphries J Cosh SM Islam MS. Factors contributing to variation in antibiotic prescribing among primary health care physicians: a systematic review. BMC Prim Care. (2024) 25:8. 10.1186/s12875-023-02223-1 38166736 PMC10759428

[7] Wu JHC Khalid F Langford BJ Beahm NP McIntyre M Schwartz KL. Community pharmacist prescribing of antimicrobials: a systematic review from an antimicrobial stewardship perspective. Can Pharm J. (2021) 154:179–92. 10.1177/1715163521999417 PMC8165883 34104272

[8] Adams AJ Weaver KK. The continuum of pharmacist prescriptive authority. Ann Pharmacother. (2016) 50:778–84. 10.1177/1060028016653608 27307413

[9] Furukawa Y Luo Y Funada S Onishi A Ostinelli E Hamza T. Optimal duration of antibiotic treatment for community-acquired pneumonia in adults: a systematic review and duration-effect meta-analysis. BMJ Open. (2023) 13(3):e061023. 10.1136/bmjopen-2022-061023 36948555 PMC10040075

[10] Johansen E Nielsen H Gillespie D Aabenhus R Hansen MP. The optimal antibiotic treatment duration for community-acquired pneumonia in adults diagnosed in general practice in Denmark (CAP-D): an open-label, pragmatic, randomised controlled trial. Trials. (2024) 25:627. 10.1186/s13063-024-08477-z 39334468 PMC11429885

[11] International Pharmaceutical Federation. Fighting Antimicrobial Resistance: The Contribution of Pharmacists. The Hague: FIP (2015).

[12] Langford BJ Morris AM. Is it time to stop counselling patients to “finish the course of antibiotics”? Can Pharm J. (2017) 150:349–50. 10.1177/1715163517735549 PMC5661683 29123590

[13] Palin V Welfare W Ashcroft DM van Staa TP. Shorter and longer courses of antibiotics for common infections and the association with reductions of infection-related complications including hospital admissions. Clin Infect Dis. (2021) 73:1805–12. 10.1093/cid/ciab159 33623985 PMC8599204

[14] Grant J Le Saux N. Duration of antibiotic therapy for common infections. J Assoc Med Microbiol Infect Dis Can. (2021) 6:181–97. 10.3138/jammi-2021-04-29 36337760 PMC9615468

[15] World Health Organization. The WHO AWaRe (Access, Watch, Reserve) Antibiotic Book. Geneva: WHO (2022).

[16] Beahm NP Smyth DJ Tsuyuki RT. Outcomes of urinary tract infection management by pharmacists (RxOUTMAP): a study of pharmacist prescribing and care in patients with uncomplicated urinary tract infections in the community. Can Pharm J. (2018) 151:305–14. 10.1177/1715163518781175 PMC6344970 31080530

[17] Mansell K Bootsman N Kuntz A Taylor J. Evaluating pharmacist prescribing for minor ailments. Int J Pharm Pract. (2015) 23:95–101. 10.1111/ijpp.12128 24930999

[18] Booth JL Mullen AB Thomson DAM Johnstone C Galbraith SJ Bryson SM. Antibiotic treatment of urinary tract infection by community pharmacists: a cross-sectional study. Br J Gen Pract. (2013) 63(609):e244–9. 10.3399/bjgp13X665206 23540480 PMC3609471

[19] Papastergiou J Trieu CR Saltmarche D Diamantouros A. Community pharmacist-directed point-of-care group A Streptococcus testing: evaluation of a Canadian program. J Am Pharm Assoc. (2018) 58:450–6. 10.1016/j.japh.2018.03.003 29681440

[20] Thornley T Marshall G Howard P Wilson APR. A feasibility service evaluation of screening and treatment of group A streptococcal pharyngitis in community pharmacies. J Antimicrob Chemother. (2016) 71:3293–9. 10.1093/jac/dkw264 27439523 PMC5079295

[21] Stewart F Caldwell G Cassells K Burton J Watson A. Building capacity in primary care: the implementation of a novel pharmacy first scheme for the management of urinary tract infection, impetigo and chronic obstructive pulmonary disease exacerbation. Prim Health Care Res Dev. (2018) 19:531–41. 10.1017/S1463423617000925 29362007 PMC6692826

[22] Hind C. NHS Grampian project: treating uncomplicated lower urinary tract infection in community pharmacy. Pharm J. (2014) 293:7837.

[23] Dyar OJ Huttner B Schouten J Pulcini C. What is antimicrobial stewardship? Clin Microbiol Infect. (2017) 23:793–8. 10.1016/j.cmi.2017.08.026 28882725

[24] Zetts RM Stoesz A Garcia AM Doctor JN Gerber JS Linder JA. Primary care physicians' attitudes and perceptions towards antibiotic resistance and outpatient antibiotic stewardship in the United States: a qualitative study. BMJ Open. (2020) 10:e034983. 10.1136/bmjopen-2019-034983 32665343 PMC7365421

[25] Klepser ME Adams AJ Klepser DG. Antimicrobial stewardship in outpatient settings: leveraging innovative physician-pharmacist collaborations to reduce antibiotic resistance. Health Secur. (2015) 13:166–73. 10.1089/hs.2014.0083 26042860

